# 肺癌干细胞Wnt信号通路的研究进展

**DOI:** 10.3779/j.issn.1009-3419.2011.08.11

**Published:** 2011-08-20

**Authors:** 小江 李, 英杰 贾, 文治 张, 莹 张, 宝乐 李, 敏娜 黄, 芳芳 包, 建国 吴, 怡 娄

**Affiliations:** 1 300193 天津，天津中医药大学第一附属医院肿瘤科 Department of Oncology, the First Teaching Hospital, Tianjin University of Traditional Chinese Medicine, Tianjin 300193, China; 2 300060 天津，天津市神经外科研 究所神经细胞实验室 Nerve Cells Laboratory, Tianjin Neurosurgery Institute, Tianjin 300060, China; 3 300193 天津，天津中医药大学 Tianjin University of Traditional Chinese Medicine, Tianjin 300193, China

**Keywords:** Wnt信号通路, 肺肿瘤, 干细胞, Wnt pathway, Lung neoplasms, Stem cells

## Abstract

Wnt信号通路在维持肺癌干细胞的增殖和克隆形成方面发挥着重要作用，可通过影响其关键蛋白质抑制肺癌干细胞增殖，为肺癌的治疗提供新的路径。本文旨在通过总结2005年-2010年肺癌干细胞及Wnt通路的研究现状，探讨Wnt通路与肺癌干细胞的关系。

## Abstract (en)

Being the most critical signaling molecule in the Wnt pathway, the Wnt/β-catenin signaling pathway plays an important role in the maintenance of the cell proliferation and clone formation of lung cancer stem cells. Since it is closely related to the WNT pathway, the proliferation of lung cancer stem cells can be restrained by blocking the WNT pathway or influencing its key protein. Such method provides a new method for the treatment of lung cancer. By summarizing the state of-the-art research of lung cancer stem cells and the Wnt pathway from 2005 to 2010, their relationship is investigated.

肺癌是全球发病率最高的恶性肿瘤，己成为人类因癌症死亡的主要原因^[1]^。近年来随着干细胞概念被引入肿瘤学的研究，以及多种肿瘤组织和癌细胞系中肿瘤干细胞得到成功分离和鉴定，“肿瘤干细胞”学说应运而生^[2]^。2007年肺癌干细胞的研究获得突破性进展，Ho等^[3]^首次发现利用Hoechst染料外排法从多种人肺癌细胞系和人肺癌临床样本中分离出的SP细胞表现出体外高致瘤率、细胞表面的ABC转运体蛋白表达上调、人端粒末端转移酶表达增高等与干细胞相似的特性，说明这部分SP细胞具有肿瘤干细胞特性。Wnt信号传导通路作为细胞信号转导系统之一，与肺癌的发生发展以及在肺癌干细胞特性维持过程中有着重要作用^[4]^。Wnt信号通路的不恰当激活，参与了肿瘤的发生及其侵袭转移的过程，该通路可能通过作用于肺癌干细胞而促使肺癌细胞复制更新或转移复发。肺癌干细胞Wnt通路的研究为肺癌治疗提供了新的突破口。

## 1 Wnt信号通路

Wnt信号通路因其启动蛋白质Wnt而得名。Wnt最初被发现为果蝇分节极性基因，其功能与胚胎发育和蜕变过程中成体翼的形成有关，Nusse等于1982年在小鼠乳头瘤病毒整合部位发现并报道了*int-1*基因，随后发现这一基因与果蝇胚胎发育基因*wingless*同源，将两者名称简并后该基因被重新命名为*Wnt*。越来越多的证据^[5-7]^表明Wnt信号途径异常激活参与了多种人类肿瘤的发生，此信号转导途径在维持肿瘤干细胞的特性如肿瘤干细胞的数量、耐药性、克隆形成能力、体内成瘤性等方面也有着重要作用。

## 2 Wnt信号转导途径

### 2.1 Wnt经典信号转导途径

即Wnt/β-catenin信号转导途径^[8]^。目前国外已在包括肺癌等多种实体瘤中检测到β-catenin的高表达^[9]^。

在没有Wnt信号刺激的情况下，β-catenin与axin/ APC、GSK-3β组成的降解复合物结合，通过磷酸化、蛋白质泛素化等过程发生降解，使胞质β-catenin保持在较低水平^[10]^。TCF/LEF与抑制物结合，阻碍下游基因的表达。Wnt信号通路启动后，Wnt配体与细胞膜表面的Fz/ LRP受体结合，β-catenin即从axin/APC、GSK-3β组成的降解复合物分离，在细胞内积聚并向细胞核内转移，与TCF/LEF结合^[11]^，从而调节下游靶基因*c-myc*、*Cyclin D1* 的表达。作为决定细胞从G0/G_1_期进入S期的开关，*c-myc* 基因不显示转录活性或呈低水平表达；只有受某些因子激活时，*c-myc*基因才大量表达^[12, 13]^，使细胞由静止期进入增殖期，促进癌细胞的增殖、侵润与转移。Cyclin D1是作用于G_1_期的重要的细胞周期蛋白，可以促进G_1_/S期转换而加速细胞周期过程。Cyclin D1经选择性剪切后可得到一种编码特殊的异构体cyclin D1b。细胞增殖与细胞凋亡相互协调，共同维持细胞数量的相对稳定，不至于细胞数量过度增减^[14]^。所以，c-myc是较早出现的Cyclin D1的异常表达，是细胞恶化的分子标志，与肺癌启动、恶性增生密切相关^[15]^。

### 2.2 Wnt-Ca^2+^途径^[16]^

Ca^2+^途径的主要过程为Wnt蛋白质结合于Fz受体，由Wnt-5a或Wnt-11激活卷曲蛋白质受体后，通过三聚体G蛋白质介导，使细胞内Ca^2+^浓度升高，从而对钙调蛋白质依赖的激酶（CaMKII）、蛋白质激酶C（PKC）起作用，活化T细胞核因子，核内NFAT的积聚导致靶基因的激活。Ca^2+^途径多由Wnt-5a等低转化型Wnt蛋白质激活，也参与原肠胚的形成，并且对于β-catenin途径起抑制作用^[17]^。

### 2.3 Wnt-细胞平面极化途径^[16]^

即PCP信号途径，Wnt蛋白质直接作用于Fz受体，使Dvl活化进而激活小三磷酸鸟苷酸酶RhoA和Rac及下游的Rho激酶和JUN激酶^[16-18]^。PCP途径主要调节细胞的迁移与极性，该途径的主要作用可能是在胚胎发育阶段调控细胞骨架的重排并参与原肠胚形成^[19]^。

## 3 Wnt信号通路与肺癌干细胞的关系

### 3.1 肺癌干细胞基因表达谱与Wnt通路的相关性

张菁茹等^[20]^采用干细胞基因芯片比较受损气管上皮在恢复24 h及48 h与正常气管上皮基因表达的差异。24 h后差异基因8个上调，31个下调；48 h后5个上调，42个下调。差异基因表达主要集中在细胞周期调控、细胞连接、FGF、BMP分子、Notch、Wnt信号通路上。该研究发现Wnt信号传导通路中APC上调、Axin下调、cyclin D1上调，提示细胞正在增殖，由G0期向S期转变。48 h后主要信号通路的基因与正常相比仍在向增殖分化方向发展，48 h后Wnt1下调意味着向分化方向发展。

罗福康等^[21]^对基因表达谱进行信号通路分析后发现，只有Wnt信号通路与肺腺癌干细胞密切相关，并且在肺腺癌干细胞与正常肺干细胞中的差异明显（*P* < 0.01）。在差异表达的基因中共有13个直接或间接参与Wnt通路信号转导，其中表达上调的有8个，下调的有5个。在表达上调的基因中TLE2达到13.1倍，而表达下调的基因的改变倍数没有超过3倍。目前未见有关TLE2与肿瘤有直接关系的报道，但基因与家族的TLE1、TLE3结构高度相似，功能基因也相近，推测它通过相似的途径来抑制肺癌干细胞的凋亡。

### 3.2 通过对关键蛋白质的影响抑制肺癌干细胞发育及增殖

作为Wn t信号通路中最关键的通道传递分子，β-catenin是一种广泛存在的多功能胞质蛋白质。研究^[22]^表明在肺干细胞中β-catenin主要通过减弱分化促进组织干细胞数量的增加，而不是促进干细胞直接增生。

肺癌干细胞的分子标记OCT-4在维持肺癌细胞的侵袭、克隆形成、耐药方面有着重要作用^[23]^。滕颖等^[24]^研究发现通过干扰A549细胞中β-catenin的表达使细胞发生G_0_-G_1_期阻滞，抑制细胞增长、克隆形成、迁移及耐药性，肺癌干细胞标志物OCT-4的基因及蛋白表达均降低；同时用10 mmol/L的GSK-3β的抑制剂LiCl作用于A549细胞，β-catenin表达增加并出现核转移，增殖活性、克隆形成能力、OCT-4表达均增强。结果提示以Wnt/β-catenin信号转导途径为靶点，抑制此通路在肺腺癌中的活化及其靶基因的表达，可能成为肺癌分子靶向治疗并有可能是针对肺癌干细胞治疗的新靶点。

Zhang等^[25]^研究发现，GATA6通过直接调控Wnt路径中的Fzd2蛋白而抑制Wnt路径。*GATA6*基因的缺失可以激活Wnt信号路径，从而能够增加细支气管肺泡干细胞的数量。PCR检测发现肺上皮细胞系MLE-15中利用siRNA对Fzd2蛋白的表达进行干预可使Fzd2蛋白表达减少大约75%，从而导致Wnt信号通路被激活，促使肺癌的发生。

癌基因*Wnt-1*和转移抑制基因*mn23-H1*同属于发育调控基因，均编码关键性调控蛋白，参与细胞的增殖、分化和凋亡。在受到异常刺激激活或发生突变表达出现异常后，又都与多种肿瘤的发生发展、侵袭和转移有关。*mn23-H1*基因可以通过阻断Wnt-catenin信号通路发挥抑制肿瘤转移作用；而*Wnt*基因又可能通过Wnt/Ca^2+^信号通路影响*mn23-H1*基因的表达^[26]^。

## 4 展望

尽管目前通过抑制Wnt信号通路而阻滞肺癌干细胞的机制尚未完全阐明，但阻断Wnt通路仍可能代表新的治疗肺癌的策略。由此衍生出多种以Wnt信号通路为靶点的抗癌治疗方法，包括以Wnt信号、β-catenin为靶点的抗癌治疗，通过蛋白质阻断阻止β-catenin表达，以β-catenin/TCF复合物的形成及其介导的转录为靶点的抗癌治疗（非甾体类抗炎药），以β-catenin信号通路下游基因为靶点的抗癌治疗，以及与Wnt/β-catenin信号通路存在协同作用的信号通路为靶点的抗癌治疗等^[27]^。

肺癌干细胞是肿瘤干细胞研究中的重要领域，对于肺癌的生长起到了至关重要的作用。Wnt信号在干细胞自我更新和增殖分化功能的保持中有着举足轻重的作用，该信号途径各组件突变的终点是干细胞区域的不适当扩增和其分化的子代的增殖，最终在获得其他变异的情况下导致恶性事件的发生，形成侵袭性的肿瘤细胞，是多种恶性肿瘤发生的重要原因。研究者在日后的研究中可通过比较分析肺癌干细胞与正常肺细胞表面分子标志的差异，分离、纯化肺癌干细胞的特异性标记，通过研究特异性标记的Wnt关键蛋白质或Wnt通路信号抑制因子阻断肿瘤干细胞的细胞周期，达到诱导肺癌细胞生长阻滞或凋亡的目的。
